# Bladder Injuries in Pediatric Age: A 12-Year Experience in a Tertiary Center

**DOI:** 10.7759/cureus.92693

**Published:** 2025-09-19

**Authors:** Carolina Soares-Aquino, Domingas Atouguia, Angélica Osório, Carlos Mariz, Miguel Campos, Leonor Carmo

**Affiliations:** 1 Pediatric Surgery, Centro Hospitalar Universitário de São João, Porto, PRT

**Keywords:** bladder injury, laparoscopy, pediatrics, trauma, ultrasound

## Abstract

Introduction: Bladder injuries are rare in pediatric age. They occur mainly after blunt trauma, frequently in motor vehicle accidents with a fastened seatbelt. When full, the bladder is more prone to rupture, and the dome is the most affected place. The common approach to intraperitoneal rupture is surgical treatment, and laparoscopic repair should be considered, although few cases have been described in pediatric age. We aim to present the cases of bladder injuries that occurred in a tertiary pediatric center in the last 12 years.

Methods: This study was conducted at the Centro Hospitalar Universitário de São João in Porto, Portugal. We performed a retrospective study including all cases of bladder injury (both extra and intraperitoneal) occurring between 2013 and 2024. We analyzed demographic data, etiology, associated lesions, clinical presentation, treatment, and follow-up.

Results: Seven cases were identified, six of them due to trauma. The diagnosis was confirmed with computerized tomography in three cases, with retrograde cystography in two cases, with ultrasound with intravesical saline in one case and intraoperatively in one case. The extraperitoneal lesions were treated conservatively, and the intraperitoneal ruptures were submitted to surgical repair, in one case by laparoscopy. No complications occurred during follow-up.

Conclusion: Although rare in pediatric age, bladder lesions can occur and have multiple causes, requiring a high level of clinical suspicion according to the mechanism of lesion and symptoms. According to our data, retrograde cystography is not mandatory, especially when other exams can give the same dynamic image. This, however, should be interpreted cautiously, given the limited sample size of this study. Laparoscopy seems to be a feasible option for diagnosis and minimally invasive treatment, especially in the setting of isolated and non-complex lesions in a stable patient.

## Introduction

Although bladder injuries are rare, the bladder represents the second most injured organ in pediatric urological trauma [1]. Bladder injury occurs in 0.05-2% of all pelvic trauma cases [2,3] and in up to 20% of pelvic fracture cases [1,4]. Up to 57% of bladder rupture cases have a concomitant pelvic fracture, although this association is much more common in adults, where a bladder injury occurs in up to 89% of pelvic fracture cases [5]. While blunt trauma is the most common cause, iatrogenic lesions during surgical procedures or acute urinary retention are other possible causes for this lesion.

Intraperitoneal rupture is more common in children than in adults [5]. A full bladder becomes located higher in the abdomen, making it more prone to intraperitoneal rupture during the traumatic event. In children, this mechanism is even more important due to a more cranial position of the bladder above the pelvic ring and a thinner abdominal wall, with less developed muscles and abdominal fatty tissue [1,5]. The dome, the bladder’s weakest and most mobile point, is the most affected place [1,3,5,6]. Extraperitoneal lesions occur in the lower parts of the bladder and are more commonly associated with pelvic fractures [5].

The symptoms of a bladder lesion are nonspecific. Suprapubic pain or tenderness, urinary retention, and hematuria are the most common [1,5]. The diagnosis requires a high level of clinical suspicion and can be difficult, especially in the setting of serious polytrauma requiring assessment of various potentially life-threatening lesions. With the improvements in imaging methods, diagnostic confirmation can be achieved with ultrasound (US) and computerized tomography (CT), which are normally performed in the evaluation of a trauma patient. Nevertheless, retrograde cystography remains the gold standard, as it evaluates bladder filling, drainage, and contrast leaks [5]. Contrast products can also be administered through the urinary catheter for the same dynamic study during US or CT imaging, obviating the need for a retrograde cystography.

Lesions can be classified according to the American Association for the Surgery of Trauma (AAST) in five grades [7]. Treatment modalities differ according to the lesion severity and can be conservative or surgical. Conservative treatment is reserved for contusions and simple extraperitoneal ruptures. While conservative treatment can be performed in selected cases of intraperitoneal rupture in adults, surgical treatment remains the standard approach in pediatric intraperitoneal ruptures. Surgery is also the preferred approach for complex extraperitoneal ruptures, either due to their size, bladder neck involvement, associated rectal or vaginal lesions, or persistent leakage after drainage [1].

Our aim was to review the cases of bladder injuries occurring in a tertiary pediatric center in the last 12 years and to describe our experience with these rare lesions, emphasizing diagnostic modalities, treatment outcomes, and the feasibility of laparoscopic repair.

## Materials and methods

We conducted a retrospective analysis of all the cases of bladder injury (traumatic and non-traumatic) diagnosed from 2013 to 2024 in the Pediatric Surgery Department of Centro Hospitalar Universitário de São João, a tertiary centre in Porto, Portugal. The research was conducted using the International Classification of Diseases codes (ICD): ICD-9 codes 596.6 and 867.0 and ICD-10 codes N32.89, S37.22XA, and S37.23XA [8,9]. Other than patient age lower than 18 years, no other inclusion or exclusion criteria were applied. The following clinical data were collected based on medical chart review: age, sex, etiology, associated lesions, exams performed, grade of the lesion according to the AAST [7] (Table 1), lesion location, treatments performed, and follow-up. All imagiological data and surgical reports were retrieved and reviewed by a single reviewer. All imagiological studies were performed by a radiologist. In the most recent case, surgical imaging was performed during the procedure. There were no ethical implications, and procedures were performed according to relevant guidelines. Continuous variables were expressed as median (minimum-maximum), and categorical variables as absolute and relative frequency.

**Table 1:** Classification of bladder injuries according to the American Association for the Surgery of Trauma. Adapted from Moore et al. [7]. Advance one grade for multiple lesions up to grade III.

Grade	Injury type	Description of injury
I	Hematoma / laceration	Contusion, intramural hematoma; partial thickness laceration
II	Laceration	Extraperitoneal bladder wall laceration <2 cm
III	Laceration	Extraperitoneal (>2 cm) or intraperitoneal (<2 cm) bladder wall laceration
IV	Laceration	Intraperitoneal bladder wall laceration
V	Laceration	Intraperitoneal or extraperitoneal bladder wall laceration extending into the bladder neck or urethral orifice (trigone)

## Results

Seven (100%) cases of bladder injury were identified in the study period. The median age was 15 years (5-17). Patients were female in four (57%) cases. The cause of the lesions was blunt trauma in six (86%) cases: four (57%) cases due to a motor vehicle accident, one (14%) bicycle accident, and one (14%) due to the fall of a bookshelf over the pelvic region. The only (14%) non-traumatic case occurred due to an iatrogenic bladder perforation while placing a suprapubic trocar for a laparoscopic appendectomy. In terms of associated lesions, the four (57%) cases due to motor vehicle accidents had pelvic fracture (4/7, 57%); hepatic contusion in one case (1/7, 14%); splenic laceration in one case (1/7, 14%); and wrist fracture and rupture of the rectus muscle in one case (1/7, 14%). The other two (29%) traumatic cases had no associated lesions.

The presenting symptoms were lower abdominal pain (7/7, 100%), gross hematuria (3/7, 43%), suprapubic bruise (1/7, 14%), and urinary retention (1/7, 14%).

**Figure 1:**
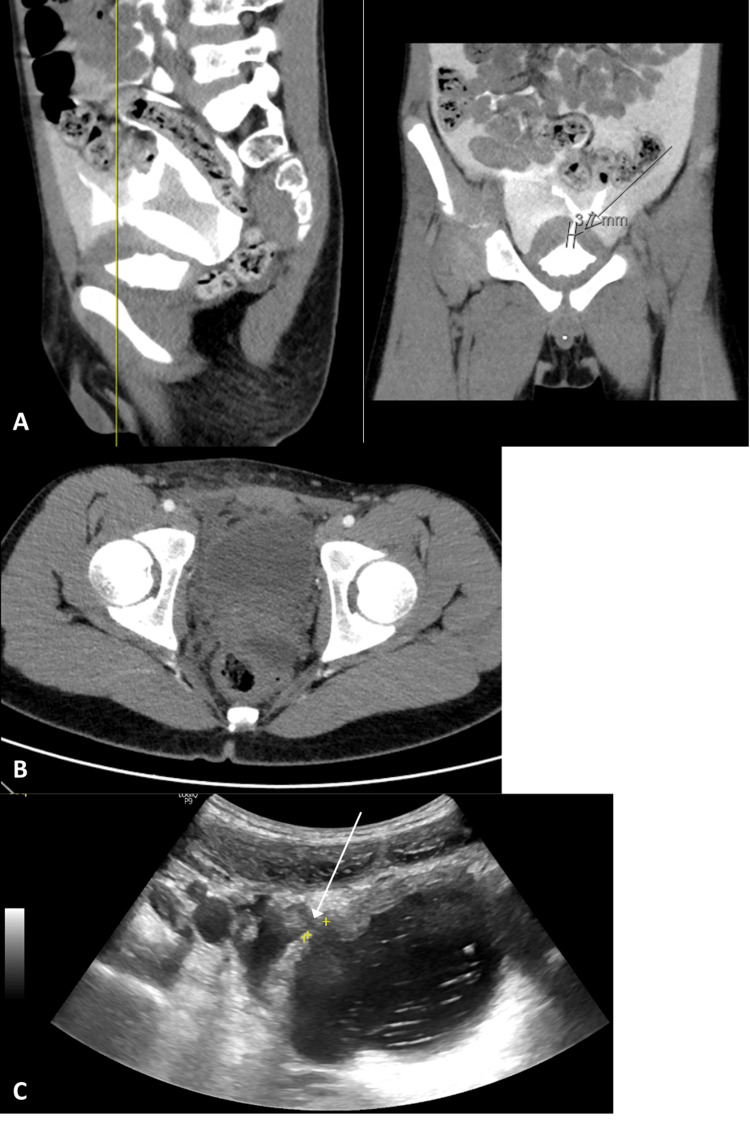
Imaging methods performed for diagnosis. (A) CT with intravesical contrast. (B) Non-diagnostic CT, without excretory phase. (C) Ultrasound with intravesical saline infusion, showing the extravasation of fluid through the rupture (arrow).

The diagnosis was confirmed with imaging modalities in six out of seven cases (85%). Abdominopelvic CT was used in four cases (57%), one of them (14%) followed by retrograde cystography. Ultrasound (US) with instillation of saline fluid through the urinary catheter was performed in three cases (43%): one (14%) followed by CT with intravesical contrast (Figure 1A), one (14%) followed by retrograde cystography, and in the last one (14%), no other exam was used for diagnosis. Interestingly, in this last case, a CT had already been performed, without the excretory phase. The rupture was not identified in this CT, but due to a high clinical suspicion (gross hematuria, suprapubic pain, and compatible traumatic mechanism), the ultrasound with intravesical saline infusion was performed (Figure 1B-1C). In the case of iatrogenic rupture after laparoscopic appendectomy, the lesion was identified on the third postoperative day, during an exploratory laparotomy performed due to suspicion of an abscess, revealing the bladder lesion.

The lesions were classified as follows: grade I (contusion) in two cases (29%), all with pelvic fracture; grade II (extraperitoneal perforation) in two cases (29%), one with associated pelvic fracture; grade III (extra and intraperitoneal perforation) in two cases (29%), one (14%) with associated pelvic fracture, the other (14%) due to the trocar placement, with no associated lesions; grade IV (intraperitoneal perforation) in one case (14%), after trauma with a bookshelf, with no associated lesions. Further information about the cases can be found in Table 2.

**Table 2:** Cases of bladder injury in our institution between 2013 and 2025. CT: computerized tomography, US: ultrasound, UTI: urinary tract infection

Case	Gender	Age (y)	Lesion classification	Etiology	Associated lesions	Lesion identification	Treatment	Urethral catheter (d)	Rupture location	Hospital discharge (d)	Complications
1	F	17	Grade I - contusion	Motor vehicle accident	Pelvic fracture; splenic laceration	Pelvic CT	Conservative	22	-	49	UTI
2	F	17	Grade I - contusion	Motor vehicle accident	Pelvic fracture; hepatic contusion	US with intra-vesical saline and retrograde cystography	Conservative	5	-	7	None
3	F	17	Grade II – extraperitoneal rupture	Motor vehicle accident	Pelvic fracture	Pelvic CT and retrograde cystography	Conservative	13	-	26	UTI
4	F	13	Grade III - extra and intraperitoneal rupture	Motor vehicle accident	Pelvic fracture	Pelvic CT	Laparotomy and detrusorraphy	13	Lateral Wall	40	None
5	M	12	Grade III - extra and intraperitoneal rupture	Iatrogenic (trocar placement)	None	US and Laparotomy (postoperative day 3)	Laparotomy and detrusorraphy	14	Dome	5	None
6	M	5	Grade IV – intraperitoneal rupture	Blunt trauma (fall of a bookshelf)	None	US with intra-vesical saline and pelvic CT with intra-vesical contrast	Laparoscopic detrusorraphy	15	Dome	8	None
7	M	15	Grade II – extraperitoneal rupture	Bicycle accident	Wrist fracture and rectus muscle rupture	US with intra-vesical saline	Conservative	10	Lateral wall	6	None

**Figure 2:**
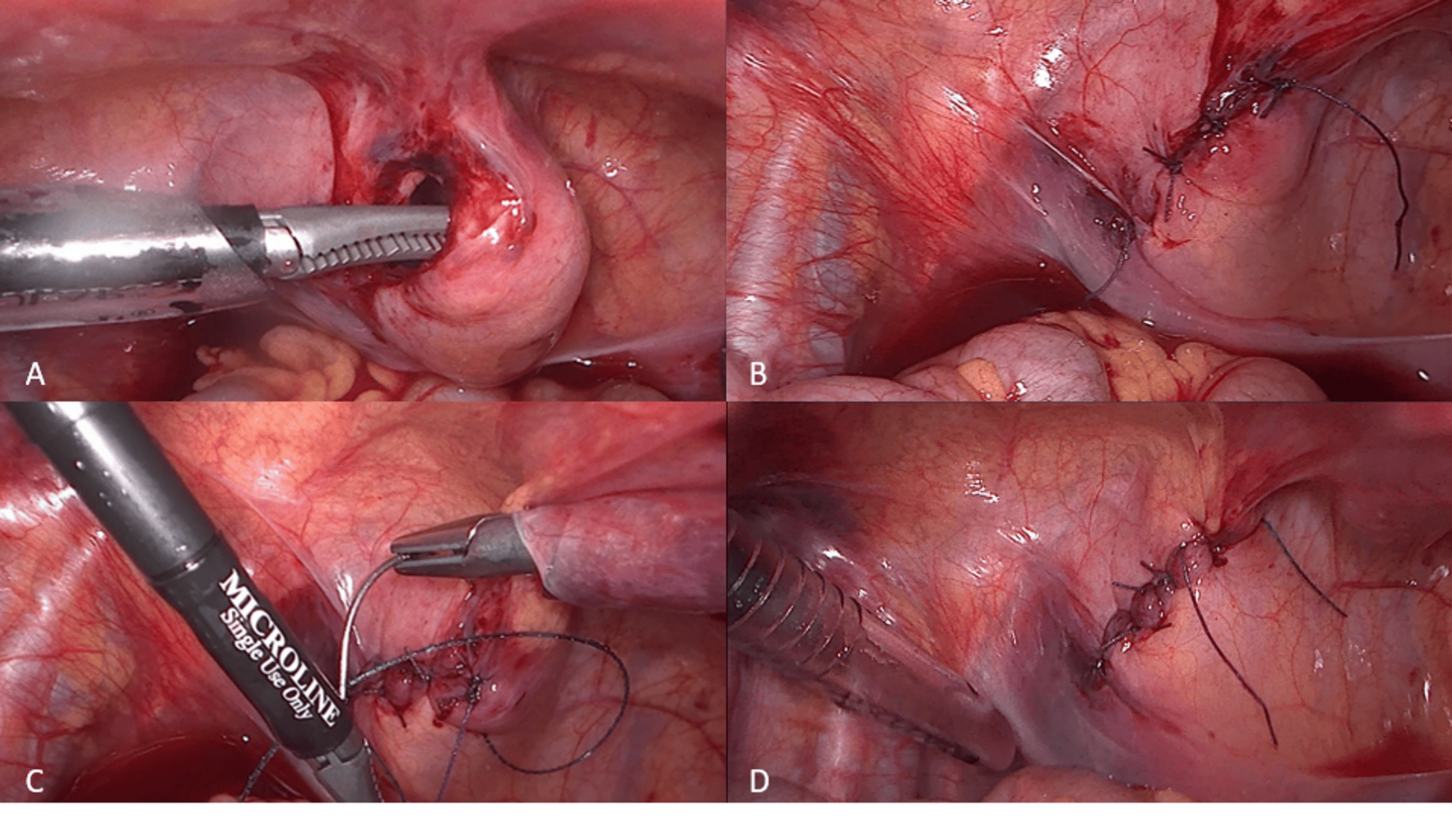
Intraoperative images of the case repaired with laparoscopy. (A) Identification of the bladder rupture. (B) First layer of interrupted sutures. (C) Second layer of suture with a knotless barbed suture thread. (D) Final overview of the repaired bladder.

The treatment was conservative in grade I and II cases (four cases, 57%), with urethral catheterization in all seven cases (100%), with a median of 13 days (5-22). In grade III and IV cases (43%), the treatment was surgical, and all were submitted to detrusorraphy. Two cases (2/3, 67%) were approached by infra-umbilical laparotomy, confirming the perforation sites to be at the lateral wall and at the dome, and a double-layer suture was performed. The last case was submitted to a laparoscopic repair, using a 10 mm umbilical port for the endoscope, and three 5 mm ports placed in the right and left lower abdominal quadrants. The perforation was identified at the bladder dome, with an extension of about 4 cm. The suture was performed in a double-layer fashion, with one layer of interrupted polyglactin sutures followed by a continuous layer of suture with a barbed knotless absorbable thread (Figure 2).

These three patients (43%) kept the urethral catheter after surgery, for a median of 14 days (13-15). An intra-abdominal drain or a drain in the Retzius space was also placed, and it was removed between postoperative days 2 and 4. No major complications occurred. There were two (29%) cases of urinary tract infection, treated with antibiotics. After a median follow-up of six months (1-6.5), no delayed complications were recorded, and all patients were well, asymptomatic, and with normal micturition.

## Discussion

Bladder injuries are rare, and their diagnosis can be difficult. In our series, the most common cause was traumatic, especially due to motor vehicle accidents, as stated in the literature [1,3]. Motor vehicle accidents, especially with high-energy forces, cause seatbelt compression and deceleration lesions that are the most common mechanisms for bladder lesions [1,3]. A pelvic fracture should always raise concern for bladder trauma, particularly when typical symptoms like hypogastric pain, hematuria, or urinary retention are present, as up to 20% of these fractures can have associated bladder lesions [2]. The non-identification and inadequate treatment of such lesions can lead to urinary ascites, infection, peritonitis, and, ultimately, death [10].

The gold standard exam for the diagnosis of these lesions is retrograde cystography [5,11]. In our series, only two patients required a retrograde cystography for lesion classification. It is important, however, to emphasize that a proper bladder filling is essential for diagnosis, as stated in the available urological trauma guidelines [12-14]. An experienced radiology team and the use of intra-vesical saline in the ultrasound or intravesical contrast in the CT provided dynamic filling and emptying images in our cases, allowing diagnosis of the lesions with the exams already performed for the management of trauma patients. To the best of our knowledge, our series is the first describing the use of ultrasound for assessment and diagnosis of these lesions in pediatric patients.

The classification of the lesion is essential for deciding the treatment modality, as most intraperitoneal lesions require surgical treatment. Extraperitoneal lesions can be managed conservatively, as long as urinary drainage is effective and there are no complex lesions [1,5]. All our cases with intraperitoneal rupture were treated surgically, in line with what is recommended in the literature. Although the pediatric guidelines only mention surgical treatment for intraperitoneal rupture (as performed in all such cases in our series), adult guidelines and case reports already suggest a possibility of conservative treatment in non-complicated intraperitoneal perforations, namely when they occur in endourologic procedures [12]. 

Deibert et al. reviewed 816 cases of bladder trauma in the United States, with only 66% of intraperitoneal bladder ruptures being surgically treated. The authors concluded that surgical treatment of intraperitoneal ruptures was associated with lower in-hospital morbidity [10].

The majority of the pediatric intraperitoneal lesions described in the literature are approached by laparotomy/open surgery. There are a few published pediatric cases of laparoscopic repair of a bladder injury [3,4]. In most of these, lesions are isolated and simple. Laparoscopy can pose some difficulties in pediatric trauma patients, namely the lack of working space and maneuverability, longer operative times, the need for pneumoperitoneum, and the learning curve, which can be extremely important in a rare entity affecting particularly trauma patients, making the decision for laparoscopic repair harder in these patients. Laparoscopy can have several advantages, namely identifying the bladder lesion, providing a good surgical field, allowing a good assessment of associated abdominal lesions, a lower rate of postoperative bowel adhesion, rapid recovery, and good cosmetic results. In the only case laparoscopically repaired in our series, no other lesions were identified, and the use of laparoscopy allowed a minimally invasive approach, complete and safe identification of the bladder wall rupture, and an effective repair. All patients kept a urethral catheter after surgery, for a median of 14 days; this is somewhat longer than the seven to 10 days of urinary diversion recommended in the literature [5]. Nevertheless, besides two cases of urinary tract infection, no other complications occurred, and all patients recovered uneventfully.

Our study has some limitations, including the small number of patients owing to the rarity of these injuries, the retrospective single-center data collection, and the limited follow-up. The heterogeneity of the diagnostic approach, namely the use of different exams for different patients, could be a source of bias and thus complicates the comparison of diagnostic accuracy.

Still, this study adds an important contribution to the current literature, where few pediatric series on bladder injury are available due to the rarity of these lesions. It also depicts one case of laparoscopically repaired bladder rupture, of which few cases are reported in the literature, as trauma patients are frequently proposed for laparotomy due to the urgent nature of the procedures. Nevertheless, laparoscopic repair has been consistently a successful strategy in stable patients, both adults and children, especially in the setting of isolated trauma [3,13-18]. It allows a minimally invasive repair of the lesion, good identification of associated lesions, fast recovery of the patient and a better cosmesis [3,15].

## Conclusions

Although rare in pediatric age, bladder lesions have multiple causes, requiring a high level of clinical suspicion according to the mechanism of the lesion and symptoms. Awareness of this type of lesion is essential. An accurate diagnosis and classification of the lesion allows the decision for surgical or conservative treatment. According to our data, retrograde cystography seems not to be mandatory, especially when other exams are performed that can be complemented with the administration of contrast or saline through the urinary catheter to give the same dynamic image to identify the lesions. This, however, should be interpreted cautiously, given the limited sample size of this study. The same applies to the use of laparoscopy in this setting. Nevertheless, laparoscopy seems to be a feasible option for diagnosis and minimally invasive treatment, especially in the setting of isolated and non-complex lesions in a stable patient.

## References

[1] Singer G, Arneitz C, Tschauner S, Castellani C, Till H (2021). Trauma in pediatric urology. Semin Pediatr Surg.

[2] Dokucu AI, Ozdemir E, Oztürk H (2000). Urogenital injuries in childhood: a strong association of bladder trauma to bowel injuries. Int Urol Nephrol.

[3] Karadag CA, Tander B, Erginel B (2016). Laparoscopic repair in children with traumatic bladder perforation. J Minim Access Surg.

[4] Bakal U, Sarac M, Tartar T, Ersoz F, Kazez A (2015). Bladder perforations in children. Niger J Clin Pract.

[5] Radmayr C, Bogaert G, Burgu B (2024). EAU Guidelines on Paediatric Urology. https://d56bochluxqnz.cloudfront.net/documents/full-guideline/EAU-Guidelines-on-Paediatric-Urology-2025.pdf.

[6] Nguyen A, Choi SJ, Gabriel B (2025). Variations and challenges in the management of traumatic bladder injuries: an experience from a large trauma center. Cureus.

[7] Moore EE, Cogbill TH, Malangoni MA (1995). Organ injury scaling. Surg Clin North Am.

[8] World Health Organization (2024). ICD-10 : international statistical classification of diseases and related health problems : tenth revision, 2nd ed. https://iris.who.int/handle/10665/42980.

[9] World Health Organization (1977). International Classification of Diseases; Manual of the International Statistical Classification of Diseases, Injuries, and Causes of Death. Alternate title, International Classification of Diseases, 1975 Revision (ICD-9). https://iris.who.int/bitstream/handle/10665/40492/9241540044_eng_v1_p1.pdf?sequence=1&isAllowed=y.

[10] Deibert CM, Glassberg KI, Spencer BA (2012). Repair of pediatric bladder rupture improves survival: results from the National Trauma Data Bank. J Pediatr Surg.

[11] Robertson-Waters E, Donaldson C, Light A, Lamb B, Thiruchelvam N (2022). Guidance for diagnosis and management of bladder injuries - is practice up to date?. BJU Int.

[12] Kitrey ND, Campos-Juanatey F, Hallscheidt P, Mayer E, Serafetinidis E, Waterloos M (2024). EAU Guidelines on Urological Trauma. https://d56bochluxqnz.cloudfront.net/documents/full-guideline/EAU-Guidelines-on-Urological-Trauma-2025.pdf.

[13] Reddy D, Laher AE, Moeng M, Adam A (2024). Bladder trauma: a guideline of the guidelines. BJU Int.

[14] Coccolini F, Moore EE, Kluger Y (2019). Kidney and uro-trauma: WSES-AAST guidelines. World J Emerg Surg.

[15] Deshpande AV, Michail P, Gera P (2017). Laparoscopic repair of intra-abdominal bladder perforation in preschool children. J Minim Access Surg.

[16] Kim C, Docimo SG (2005). Use of laparoscopy in pediatric urology. Rev Urol.

[17] Creswell M, Laird C, Juang D, Bowlin P (2024). Laparoscopic Cystorrhaphy in a pediatric patient with a penetrating bladder injury. J Urol.

[18] Kim B, Roberts M (2012). Laparoscopic repair of traumatic intraperitoneal bladder rupture: case report and review of the literature. Can Urol Assoc J.

